# The Clinical Spectrum of Kommerell’s Diverticulum in Adults with a Right-Sided Aortic Arch: A Case Series and Literature Overview

**DOI:** 10.3390/jcdd8030025

**Published:** 2021-02-26

**Authors:** Philippe J. van Rosendael, J. Lauran Stöger, Philippine Kiès, Hubert W. Vliegen, Mark G. Hazekamp, David R. Koolbergen, Hildo J. Lamb, Monique R. M. Jongbloed, Anastasia D. Egorova

**Affiliations:** 1 Center for Congenital Heart Disease Amsterdam-Leiden (CAHAL), Leiden University Medical Center, 2333 ZA Leiden, The Netherlands; p.j.van_rosendael@lumc.nl (P.J.v.R.); p.kies@lumc.nl (P.K.); H.W.Vliegen@lumc.nl (H.W.V.); M.G.Hazekamp@lumc.nl (M.G.H.); D.R.Koolbergen@lumc.nl (D.R.K.); M.R.M.Jongbloed@lumc.nl (M.R.M.J.); 2 Department of Cardiology, Heart Lung Centre, Leiden University Medical Centre, 2333 ZC Leiden, The Netherlands; 3 Department of Radiology, Leiden University Medical Center, 2333 ZA Leiden, The Netherlands; J.L.Stoger@lumc.nl (J.L.S.); h.j.lamb@lumc.nl (H.J.L.); 4 Department of Cardiothoracic Surgery, Leiden University Medical Center, 2333 ZC Leiden, The Netherlands; 5 Department of Anatomy and Embryology, Leiden University Medical Center, 2333 ZC Leiden, The Netherlands

**Keywords:** Kommerell’s diverticulum, right sided aortic arch, anomalous left subclavian artery, arteria lusoria, tracheal compression, esophageal compression

## Abstract

Background: Kommerell’s diverticulum is a rare vascular anomaly characterized as an outpouch at the onset of an aberrant subclavian artery. In the variant of a right-sided aortic arch, the trachea and esophagus are enclosed dorsally by the arch. In the configuration of an aberrant left subclavian artery, a Kommerell’s diverticulum and persisting ductus arteriosus or ductal ligament enclose the lateral side, forming a vascular ring which may result in (symptomatic) esophageal or tracheal compression. Spontaneous rupture of an aneurysmatic Kommerell’s diverticulum has also been reported. Due to the rarity of this condition and underreporting in the literature, the clinical implications of a Kommerell’s diverticulum are not well defined. Case summary: We describe seven consecutive adult patients with a right-sided aortic arch and an aberrant course of the left subclavian artery (arteria lusoria), and a Kommerell’s diverticulum, diagnosed in our tertiary hospital. One patient had severe symptoms related to the Kommerell’s diverticulum and underwent surgical repair. In total, two of the patients experienced mild non-limiting dyspnea complaints and in four patients the Kommerell’s diverticulum was incidentally documented on a computed tomography (CT) scan acquired for a different indication. The size of the Kommerell’s diverticulum ranged from 19 × 21 mm to 30 × 29 mm. In the six patients that did not undergo surgery, a strategy of periodic follow-up with structural imaging was pursued. No significant growth of the Kommerell’s diverticulum was observed and none of the patients experienced an acute aortic syndrome to date. Discussion: Kommerell’s diverticulum in the setting of a right-sided aortic arch with an aberrant left subclavian artery is frequently associated with tracheal and esophageal compression and this may result in a varying range of symptoms. Guidelines on management of Kommerell’s diverticulum are currently lacking. This case series and literature overview suggests that serial follow-up is warranted in adult patients with a Kommerell’s diverticulum with small dimensions and no symptoms, however, that surgical intervention should be considered when patients become symptomatic or when the diameter exceeds 30 mm in the absence of symptoms.

## 1. Introduction: From Embryology to Clinical Presentation

A Kommerell’s diverticulum is a rare vascular anomaly and is characterized by an aneurysmatic onset of an aberrant left or right subclavian artery [1]. A Kommerell’s diverticulum was first described by the radiologist Burckhard Kommerell in 1936 in a patient with dysphagia due to esophageal compression that was related to an aneurysmatic onset of an aberrant right subclavian artery from a left-sided aortic arch [2,3]. Currently, the definition also includes an aneurysmatic origin of an aberrant left subclavian artery from a right-sided aortic arch, and it may also be present in patients with a double aortic arch [4]. From surgical and radiology series, it has been estimated that the prevalence of a left-sided aortic arch with an aberrant right subclavian artery ranges between 0.7–2.0% and between 0.04–0.4% for a right-sided aortic arch with an aberrant left subclavian artery [3,5]. Overall, it is reported that 20–60% of the aberrant subclavian arteries are associated with a Kommerell’s diverticulum, defined as an offset diameter being more than 50% larger than the diameter of the distal segment of the aberrant subclavian artery [3,5,6]. Recently, Erben et al. reported a retrospective analysis of the radiological database of Yale from the years 1999 to 2006. Of the 75 patients with a Kommerell’s diverticulum 63% had a left-sided arch with aberrant right subclavian artery and 37% had a right-sided arch with left subclavian artery [7]. In contrast, a retrospective analysis of the radiology database of the Mayo clinic of the years 1990 to 2014 by Poterucha et al. (Congress abstract data, American Cardiology College 2015) showed that in 863 patients who were identified with an aberrant subclavian artery, that a Kommerell’s diverticulum was observed in 14% (*n* = 121), and of those patients 60% (*n* = 73) had a right-sided aortic arch and aberrant left subclavian artery [8]. The discrepancies between the data of Erben et al. and Poterucha et al. may in part be explained by differences in the used criteria to define a Kommerell’s diverticulum or due to diagnostic underreporting.

**Figure 1:**
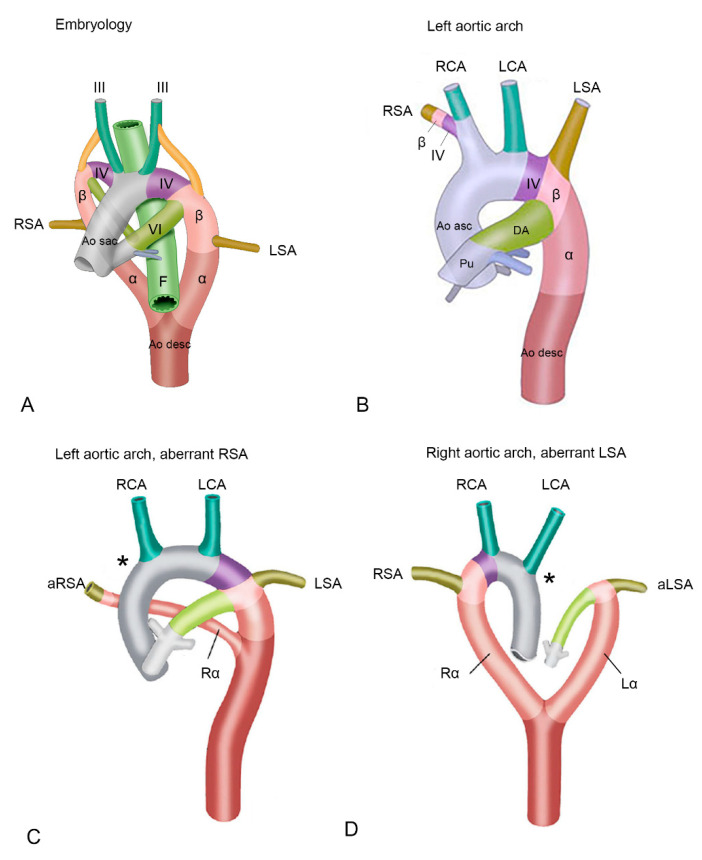
(**A**) Schematic overview of early development. Embryological segments are superimposed on the developing vascular system of the cardiac outflow tract. The vascular system evolves from an initially almost symmetrical bilateral system, into an asymmetrical system. Structures relevant to development of the outflow tract arteries are indicated. (**B**) Left sided aortic arch. The right 4th pharyngeal arch artery (PAA) and beta-segment now form the base of the right subclavian artery, connecting it to the aorta via the brachiocephalic artery. The right alpha-segment has regressed. (**C**) Left aortic arch with an aberrant right subclavian artery. Due to deficiency of the right 4th PAA (asterisk *), the proximal part of the right subclavian artery is deficient, and an aberrant right subclavian artery is connected distally to the aorta descendens via a persisting right alpha-segment. (**D**) Right aortic arch with an aberrant right subclavian artery. In this case, the left 4th PAA is deficient (asterisk *) and the aberrant left subclavian artery is connected to the aorta descendens via a persisting left alpha-segment. Abbreviations in the figure: α, alpha-segment; aLSA, aberrant left subclavian artery; Ao desc, descending aorta; Ao sac, aortic sac; aRSA, aberrant right subclavian artery; β, beta-segment; DA, ductus arteriosus; F, foregut (will later form a.o. esophagus and trachea); Lα, left alpha-segment, LCA, left carotid artery; LSA, left subclavian artery; Pu, pulmonary trunk; Rα, right alpha-segment; RCA, right carotid artery; RSA, right subclavian artery. III, IV and VI, 3rd, 4th and 6th pharyngeal arch arteries (PAA) respectively. Color coding: Grey, ascending aorta; green, carotid arteries, derived form 3rd PAA; purple, aortic B segment (in between carotid and subclavian artery, derived from 4th PAA, olive green, left subclavian arteries (derived from 7th intersegmental arteries); dark brown, descending aorta; dark pink, alpha-segment; light pink, beta-segment. Figures are modified with permission from Molin et al. Cardiovasc Res. 2002 [11] and Molin et.al Birth Defects Res A Clin Mol Teratol. 2004 [12].

From an embryological perspective and based on extensive studies in animal models, a Kommerell’s diverticulum has been suggested to result from incomplete regression of the 4th pharyngeal arch artery (PAA) [6,9,10]. The embryological vascular system is initially bilateral, and both a right and left-sided aortic arch can be identified (Figure 1A). In the right and left aortic arches several different segments can be recognized (Figure 1A), that are partly derived from the PAAs, embryological bilateral vascular structures that during early development arise from the aortic sac, the first part of the developing aorta that can be recognised. Part of the left and right aortic arches are derived from the right and the left 4th PAA [9,10,11,12]. The right and left 4th PAAs give rise to the aortic segment in between the carotid and subclavian arteries (also referred to as the aortic B segment) and connect distally via so-called beta- and alpha-segments to the descending aorta (Figure 1A) [9,10,11,12].

During development of a normal left aortic arch (Figure 1B), the right alpha-segment (i.e., the part connecting the right arch to the descending aorta, after the offset of the right common carotid and the right subclavian artery) regresses [10]. In this case the left 4th PAA will persist to form the normal left aortic arch, that is connected via the left beta- and alpha-segment to the descending aorta. The aortic arch tributaries are also largely derived from the embryonic PAAs. The left and right third PAA will contribute to the left and right common carotid arteries. The subclavian arteries are derived from the 7th intersegmental arteries, and will eventually connect to the aortic arch at the level of the beta-segment (Figure 1A,B). During normal development, the right 4th PAA and beta-segment will eventually form the origin of the right subclavian artery (Figure 1B), connecting it to the aorta via the brachiocephalic trunk [13]. If, however, the right 4th PAA shows an abnormal regression, this connection cannot be established resulting in an aberrant, more distal origin of the right subclavian artery from the descending aorta, via a persisting right alpha-segment [3] (Figure 1C). This aberrant subclavian artery courses posteriorly to the esophagus and trachea, to supply the right arm [9,10].

**Figure 2:**
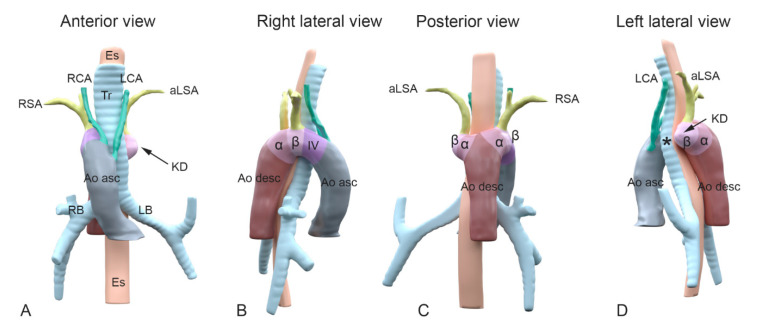
A schematic representation of the embryological aortic segments involved in development of a Kommerell’s diverticulum. Panel (**A**–**D**): Different views of 3D reconstructions based on computed tomography angiography (CTA) images of a patient with a right-sided aortic arch and an aberrant left subclavian artery. Colors superimposed on the figures indicate segments derived from the embryological pharyngeal arch arteries (PAA) and other embryological aortic segments. Color coding: Grey: ascending aorta; green: carotid arteries, derived from 3rd PAA; purple: aortic B segment (in between carotid and subclavian artery, derived from 4th PAA); olive green: subclavian arteries (derived from 7th intersegmental arteries); dark brown: descending aorta; dark pink: alpha-segment; light pink: beta-segment. Colors and segments are derived from Molin et al. Cardiovasc Res. 2002 [11], and Molin et al. Birth Defects Res A Clin Mol Teratol. 2004 [12]. (**A**) Anterior view. Note the right-sided position of the arch in relation to trachea and esophagus. Upstream in the aorta, the following aortic arch tributaries can be encountered: left carotid artery (LCA), right carotid artery (RCA), right subclavian artery (RSA) and an aberrant left subclavian artery (aLSA). (**B**) Right lateral view indicating the embryonic aortic segments of the right aortic arch. (**C**) Posterior view and (**D**) left lateral view. The aLSA connects to a dilated embryonic beta-segment (arrow), that forms the base of a Kommerell’s diverticulum (KD). The asteriks * indicates where the location of the left 4th PAA (that is missing here) would have been in case of a double aortic arch. As the 4th PAA borders the beta-segment, the the segment indicating the dilated beta-segment, may also comprisee part of an incompletely regressed left 4th PAA. α, alpha-segment; Ao asc, ascending aorta; Ao desc, descending aorta; aLSA, aberrant left subclavian artery; β, beta-segment; Es, esophagus; LB, left bronchus; LCA, left carotid artery; LSA, left subclavian artery; KD, Komerell’s diverticulum; RB, right bronchus; RCA, right carotid artery; RSA, right subclavian artery; Tr, trachea.

In case of development of a right (instead of a left) aortic arch, a complete mirror image of the arch arteries can be encountered: the right arch first gives rise to a left brachiocephalic trunk, and subsequently a right carotid and right subclavian artery arising directly from the aortic arch. The right aortic arch in this case connects to the descending aorta via a persisting right alpha-segment, whereas the left alpha-segment has regressed. If in case of a right aortic arch, the left 4th PAA (partially) regresses, an aberrant left subclavian artery will connect to the descending aorta via a persisting left alpha-segment, and will course dorsally to the esophagus and trachea to supply the left arm [9,10] (Figure 1D). One could hypothesize that a Kommerell’s diverticulum results from an only partial regression of the 4th PAA, thus still forming part of the base of the left subclavian artery together with the beta-segment, but lacking a connection to the proximal part of the aorta (Figure 2). The arterial duct connects to the aorta in this area, and will thus complete the vascular ring formed by the right aortic arch. After closure of the duct, traction by the arterial ligament is considered to be an important factor in growth of the Kommerell’s diverticulum.

The configuration of a right-sided aortic arch and left-sided aberrant subclavian artery in the presence of a Kommerell’s diverticulum and ductal ligament (or persistent ductus arteriosus) forms a vascular ring around the esophagus and trachea. This may result in symptoms related to esophageal and tracheal compression, depending on location and degree of compression. For the trachea of an adult, it is estimated that exertional dyspnea occurs when the tracheal luminal diameter is less than 8mm and that symptoms in rest occur when the diameter is less than 6 mm [14]. The progression to symptomatic esophageal and tracheal compression varies and may partly explain the wide spectrum of clinical manifestations of a Kommerell’s diverticulum, that range from severely symptomatic cases requiring surgical intervention during the early years of life, to an incidental finding in asymptomatic adult patients. The latter type is probably underreported in literature. Additionally, Kommerell’s diverticulum may be associated with an increased risk of acute aortic/vascular syndromes. Cina and colleagues reported an aortic dissection rate ranging between 19 to 53% in the 32 case reports available at time of publishing [15]. However, this rate seems inconsistent with clinical experience and is likely based on significant (over) reporting bias [7].

As there is currently no clear consensus with regards to the clinical management of Kommerell’s diverticulum or defined indicators to justify surgery, we discuss the clinical presentation, diagnosis and management, and follow-up of 7 consecutive patients from our academic referral center. The current series explores the relation between the anatomy and the occurrence and severity of symptoms in patients with a right-sided aortic arch, left aberrant subclavian artery and a Kommerell’s diverticulum considering this configuration as a distinct entity rather than also including patients with a left-sided aortic arch.

## 2. Case Presentation

### 2.1. Patient 1

**Figure 3:**
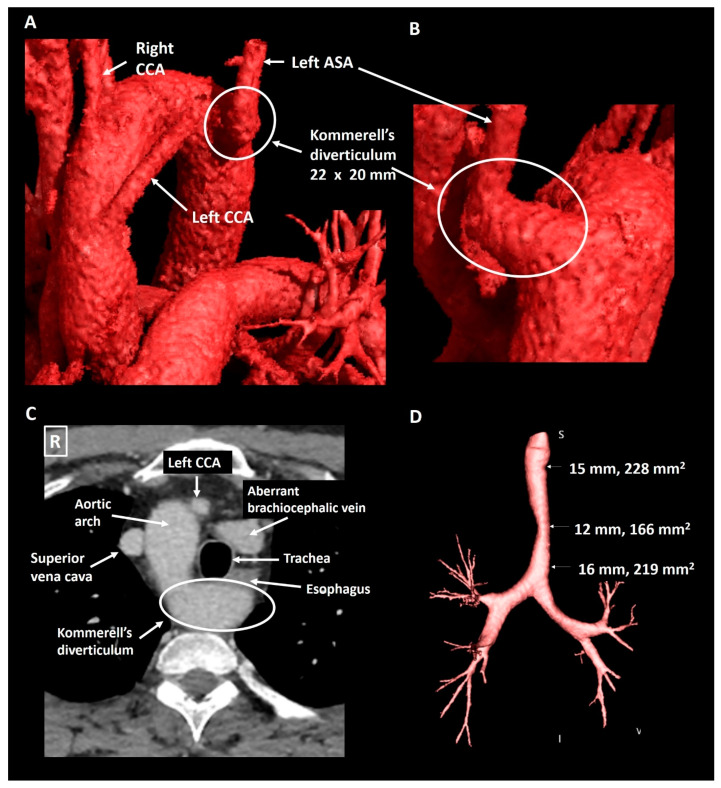
Patient 1. Panel (**A**) shows a CT-image reconstructed with global illumination rendering (iGIR) (Vitrea^®^, Vital Images) in a anterolateral orientation depicting a right-sided aortic arch with an aberrant left subclavian artery. Panel (**B**) shows a close up of the oblique posterior view of a Kommerell’s diverticulum as the onset of the aberrant left subclavian artery. Panel (**C**) is an axial reconstruction depicting the anatomical relationship between the aortic arch, Kommerell’s diverticulum and an aberrant brachiocephalic vein which encircle the trachea resulting in mild compression. Panel (**D**) is a minimum intensity projection of the trachea showing mild external compression. ASA, aberrant subclavian artery; CCA, common carotid artery.

A 40-year old male patient was evaluated at the cardiology outpatient clinic prior to renal transplantation. His history included Ewing’s sarcoma, left nephrectomy due to metastatic disease five years earlier and subsequent right kidney failure. The initial Computed Tomography (CT) Angiography (CTA, Figure 3) showed a Kommerell’s diverticulum of 22 × 20 mm (319 mm^2^) from a right-sided aortic arch with aberrant left subclavian artery. In addition, the brachiocephalic vein had an aberrant course ventral to the trachea and in this case, this structure contributed to the mild tracheal compression. The ductus arteriosus had regressed (ductal ligament) leading to an incomplete vascular ring. The patient experienced no pulmonary complaints. Echocardiography showed normal biventricular function and no hemodynamically significant valvular disease. Scintigraphy revealed reversible ischemia of the anterior segments and coronary angiography confirmed a significant stenosis in the proximal left anterior descending artery, which was treated with percutaneous coronary intervention and stenting. Patient routinely undergoes chest and abdominal CT surveillance to exclude recurrence of malignant disease. His most recent exam, performed five years after initial documentation of the Kommerell’s diverticulum, showed stable dimensions of the vascular structures.

### 2.2. Patient 2

**Figure 4:**
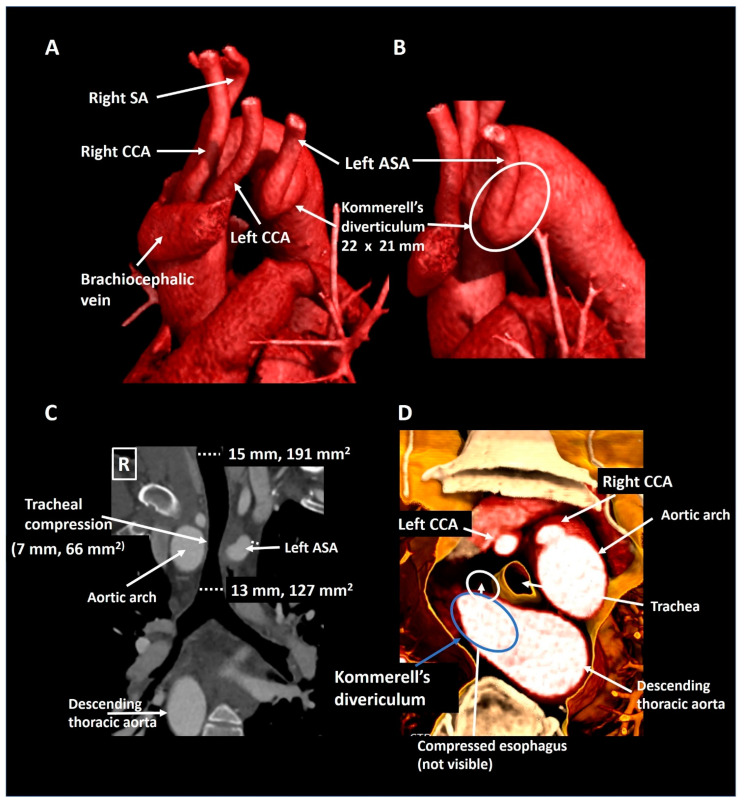
Patient 2. Panel (**A**) shows a lateral computed tomography reconstruction depicting a right-sided aortic arch with an aberrant left subclavian artery. The zoomed posterolateral reconstruction in panel (**B**) shows that the aberrant left subclavian artery is arising from a Kommerell’s diverticulum. Panel (**C**) is a coronal reconstruction that reveals tracheal compression. The axial 3D cross-section (panel (**D**)) shows partial posterior compression of the trachea which was secondary to tracheal bowing and not directly to compression by the vascular structures. ASA, aberrant subclavian artery; CCA, common carotid artery; SA, subclavian artery.

A 40-year old male presented to the emergency department with partial paraplegia due to multiple traumatic cervical fractures after an accident. As an incidental finding, the CTA (Figure 4) showed a right-sided aortic arch with an aberrant left subclavian artery and a Kommerell’s diverticulum of 22 × 21 mm (357 mm^2^) with partial compression of the trachea due to bowing of the posterior tracheal membrane. This configuration formed an incomplete vascular ring. He experienced no complaints related to the Kommerell’s diverticulum, hence a watchful waiting strategy is pursued with periodic CTA follow-up.

### 2.3. Patient 3

**Figure 5:**
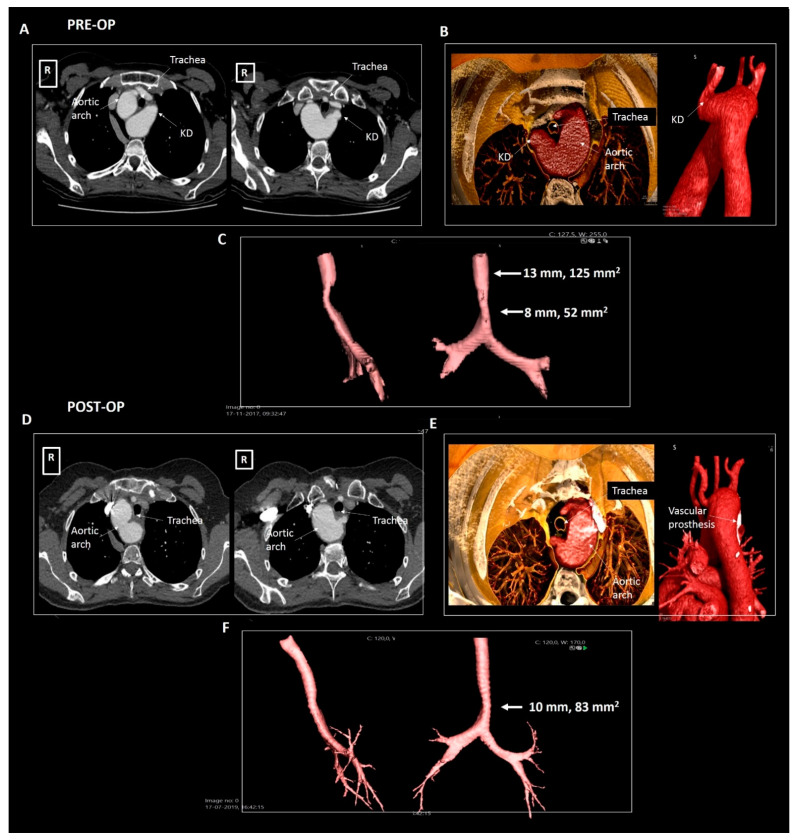
Patient 3. Panels (**A**–**C**) pre-operative imaging, panels (**D**–**F**) post-operative imaging. Panel (**A**) shows the axial reconstructions illustrating a right-sided aortic arch, aberrant left subclavian artery and a Kommerell’s diverticulum compressing the trachea and esophagus. Panel (**B**) shows the volume rendering images and panel (**C**) depicts the compressed trachea. Panel (**D**) shows the post-operative result after surgical resection of the Kommerell’s diverticulum in axial reconstructions and panel (**E**) in volume rendered reconstructions. Panel (**F**) depicts the trachea, illustrating some increase in tracheal dimensions. KD, Kommerell’s diverticulum.

A 50-year old woman was referred by the pulmonologist with progressive symptoms of dysphagia since the last three years. The forced expiratory volume during spirometry was reduced (2.76 L, 90% of predicted) and she had recently developed an inspiratory stridor. Chest X-ray revealed a right-sided aortic arch. Further investigation with a CTA (Figure 5) showed an aberrant left subclavian artery with a Kommerell’s diverticulum of 30 × 29 mm. At the level of the Kommerell’s diverticulum, there was significant tracheal (transverse luminal diameter, 7 mm) and esophageal compression (without any upper gastrointestinal tract complaints). The patient underwent surgical resection of the Kommerell’s diverticulum with the subsequent placement of a Vascutek prosthetic aortic patch and re-implantation of the left subclavian artery within a branch of the patch. The surgery was uneventful and patient’s symptoms resolved. Postoperatively the patient developed mild symptoms of vocal cord dysfunction, which improved after speech therapy. Follow-up CTA after 6 and 12 months showed recovery of the tracheal (transverse luminal diameter 10 mm) and esophageal dimensions. The patient is under periodic follow-up.

### 2.4. Patient 4

**Figure 6:**
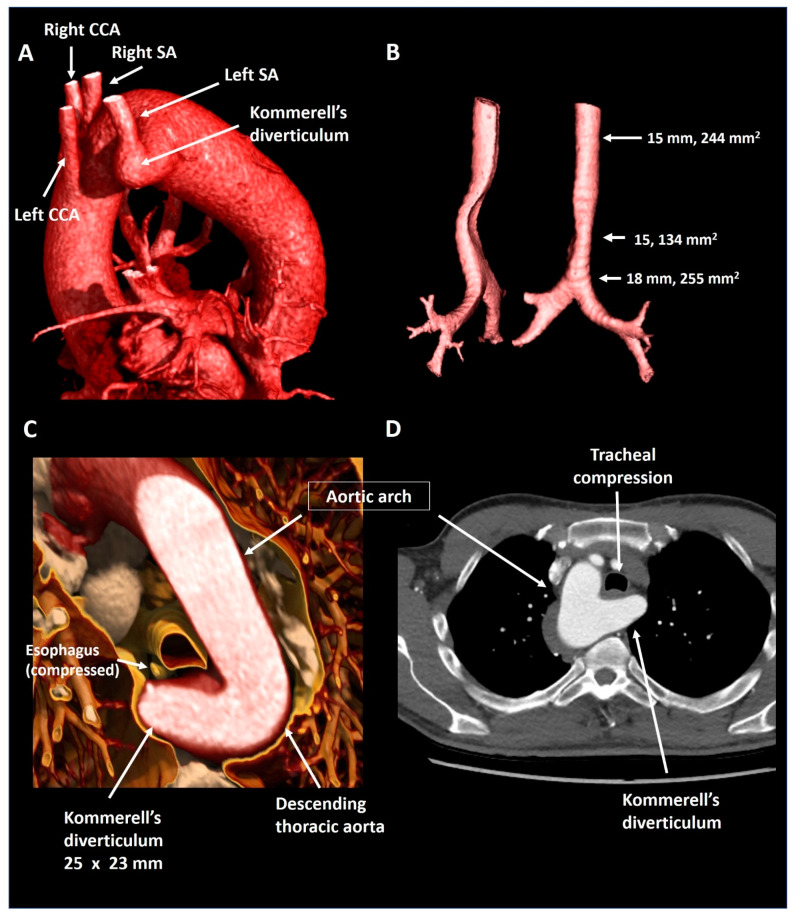
Patient 4. Panel (**A**) shows a lateral reconstruction depicting a right-sided aorta and an aberrant left subclavian artery with Kommerell’s diverticulum. Panel (**B**) shows the tracheal compression in isolation. Panel (**C**) provides a volume rendered axial reconstruction and panel (**D**) a standard axial reconstruction. CCA, common carotid artery; SA, subclavian artery.

A 55-year old male was evaluated by his general practitioner due to mild complaints of dyspnea upon exertion, whilst training for a triathlon. A chest X-ray showing a right-sided aorta and a reduced maximal expiratory peak flow during spirometry testing prompted further investigation. Subsequent CTA (Figure 6) showed an aberrant left subclavian artery with a Kommerell’s diverticulum of 25 × 23 mm (575 mm^2^) that caused mild tracheal compression in the absence of a persisting ductus arteriosus. As Kommerell’s diverticulum carries a risk of rupture, caution was advised with regard to peak and maximal exercise efforts. CTA was repeated 1 year after diagnosis, revealing stable dimensions of the Kommerell’s diverticulum. The patient is under periodic follow-up.

### 2.5. Patient 5

**Figure 7:**
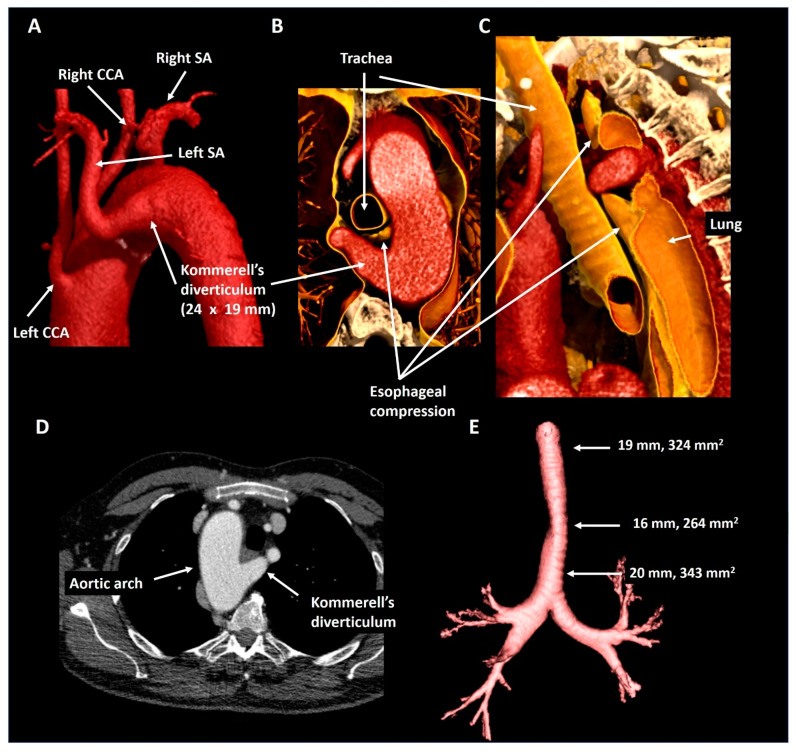
Patient 5. Panel (**A**) shows a posterolateral view depicting a right-sided aortic arch and an aberrant left subclavian artery with a Kommerell’s diverticulum. The axial (panel (**B**)) and oblique sagittal (panel (**C**)) lateral 3D volume rendering reconstructions reveal that the Kommerell’s diverticulum causes esophageal compression. Panel (**D**) shows the axial reconstruction and in panel (**E**) the mild tracheal compression is viewed in isolation. CCA, common carotid artery; SA, subclavian artery.

A 63-year old male underwent curative surgery for colorectal carcinoma with hepatic metastases and underwent 6-monthly CT follow-up to screen for potential recurrence of malignancy. The first chest CT (Figure 7) revealed a right-sided aortic arch and an aberrant left subclavian artery arising from a Kommerell’s diverticulum of 24 × 19 mm (343 mm^2^) that resulted in asymptomatic esophageal compression. Given the lack of symptoms and known metastatic disease, it was decided to refrain from intervention and opt for CTA follow-up. During the subsequent 3 years, diverticular dimensions have remained stable.

### 2.6. Patient 6

**Figure 8:**
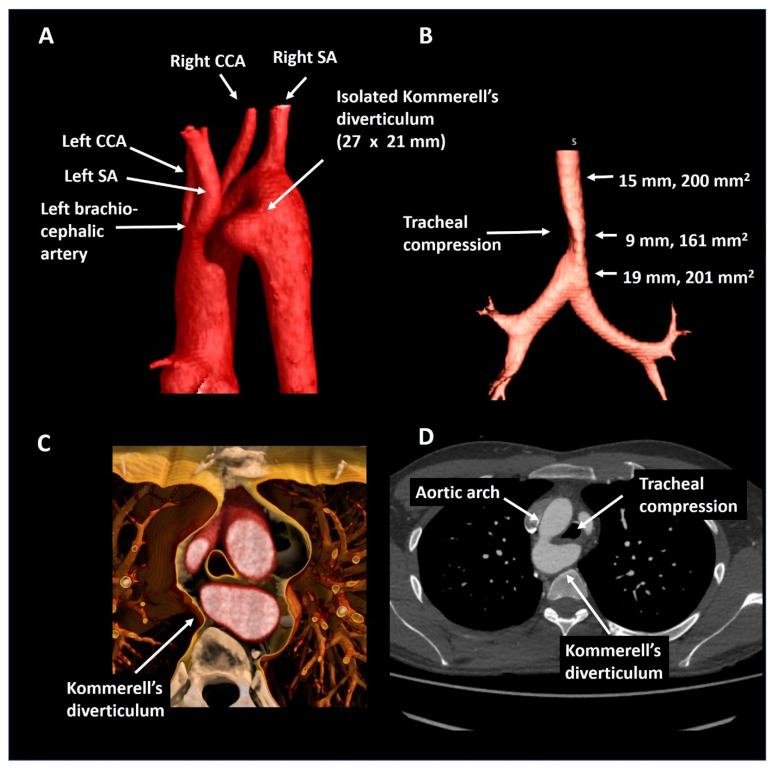
Patient 6. Panel (**A**) shows a posterolateral view of a right-sided aortic arch with mirror imaging branching and an isolated Kommerell’s diverticulum compressing the trachea (panel (**B**)) by the formation of a vascular ring (panel (**C**)). Panel (**D**) shows the axial reconstruction. CCA, common carotid artery; SA, subclavian artery.

A 23-year old male who was referred by a general practitioner after analysis for suspected asthma revealed a right-sided aortic arch on a chest X-ray. Subsequent CTA (Figure 8) showed mirror image branching of the aortic arch arteries and an isolated Kommerell’s diverticulum of 27 × 21 mm (440 mm^2^), likely due to traction of an arterial ligament. This anomaly resulted in mild tracheal and esophageal compression. As the patient had an excellent exercise capacity (375 Watt at bicycle exercise testing) after the initiation of bronchodilator therapy, and a relatively small size of Kommerell’s diverticulum, a strategy of watchful waiting with periodic CTA follow-up was pursued. In addition, caution was advised with regards to peak and maximal exercise efforts. The patient is under periodic follow-up.

### 2.7. Patient 7

**Figure 9:**
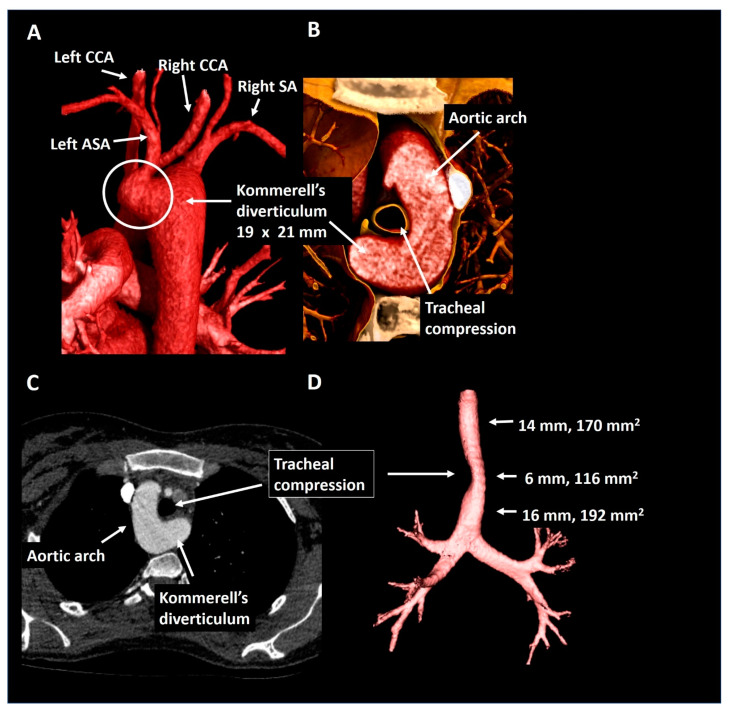
Patient 7. Panel (**A**) shows a posterior view of the descending thoracic aorta, right-sided aortic arch and Kommerell’s diverticulum as offset for an aberrant left subclavian artery. In panel (**B**) the short axis configuration is depicted showing mild tracheal compression. Panel (**C**) shows the axial reconstruction and panel (**D**) provides the trachea in isolation. ASA, aberrant subclavian artery; CCA, common carotid artery; SA, subclavian artery.

A 25-year old female with the Noonan syndrome and a right-sided aortic arch, was referred for cardiac screening due to an active pregnancy wish. Echocardiography showed a structurally normal heart. For better visualization of the thoracic aorta she underwent CTA (Figure 9) that confirmed the right-sided aortic arch and showed a left aberrant subclavian artery and a Kommerell’s diverticulum of 19 × 21 mm. The trachea and esophagus were only mildly compressed. In the absence of symptoms and a good exercise capacity (160 Watt at bicycle exercise testing) a watchful waiting strategy was pursued and the patient was advised to avoid maximal exercise. The patient is under periodic follow-up.

## 3. Discussion: Clinical Implications

Due to its incidental nature and presumed underreporting in the literature, the natural history and exact clinical consequences of Kommerell’s diverticulum are unknown. Consequently, there are currently no guidelines that specifically address how patients with Kommerell’s diverticulum should be managed. We report our experience with 7 consecutive adult patients with the anatomic variant of an aberrant left subclavian artery with Kommerell’s diverticulum and a right-sided aortic arch.

From the current case-series (Table 1), 1 of the 7 patients had significant symptoms related to extrinsic esophageal and tracheal compression and underwent successful surgical repair. The Kommerell’s diverticulum of the operated patient (patient 3) was the largest of the current series (30 mm). Nonetheless, it should be appreciated that Kommerell’s diverticulum is frequently an incidental finding. In total, six patients from the current series had only slight esophageal or tracheal compression on CT that was not associated with significant symptoms and in these patients a strategy of watchful waiting with serial CTA follow-up was pursued. Bearing in mind the risk of acute complications, we advised these patients to avoid peak exercise to reduce the shear stress and vascular strain on the diverticulum.

Colleagues from the Radiology department of the Yale University showed that the majority (63%) of 75 patients with Kommerell’s diverticulum had a left-sided aortic arch [7], whereas in the 121 patients reported by Poterucha et al. [8] 60% had a right-sided aortic arch. Bogerijen et al. observed a left-sided aortic arch in 73% of a surgical cohort of 22 patients [1]. However, data from larger populations is lacking, particularly from individuals without symptoms or surgical intervention. The combination of Kommerell’s diverticulum, an aberrant left subclavian artery, a right-sided aortic arch and persistent arterial duct may form a vascular ring around the trachea and esophagus. Depending on the size of the diverticulum, the local anatomy and presence of concomitant (vascular) anomalies, this variant may more frequently result in symptoms than the combination of a Kommerell’s diverticulum and an aberrant right subclavian artery which has a retroesophageal course. Nowadays, non-invasive 3D imaging techniques such as CT or magnetic resonance have largely replaced the need for bronchoscopy or barium swallow testing in adult patients [3].

Some groups advocate an aggressive intervention strategy in patients with Kommerell’s diverticulum based on the reported high rupture rate of up to 53% [15]. Kim and colleagues recommend surgical resection of a Kommerell’s diverticulum even in asymptomatic patients and reported the retrospective results of 19 adult patients who underwent surgical correction independent of the size of the Kommerell’s diverticulum, that ranged from 15–45 mm [16]. In this series, the surgical procedure was complicated by an perioperative type A dissection (5.3%), laryngeal and phrenic nerve injury (10.5%), and transient neurologic dysfunction (5.3%). Patient 3 in our series had mild symptoms of vocal cord dysfunction due to perioperative laryngeal nerve injury. Other single centers’ recommendations include an indication for surgery when the Kommerell’s diverticular dimension exceeds 50 mm in the long-axis [17] or if the maximal orifice diameter exceeds 30 mm [18]. In our center, we consider operative treatment in adult patients with a diverticular orifice diameter of 30 mm or more due to the risk of dissection or rupture, irrespective of complaints. The lack of international consensus and the periprocedural risks advocate the importance of anatomical and clinical criteria with regards to patient selection for surgical management in adults.

There may be factors beyond the absolute diverticular size when it comes to risk of dissection or rupture. A recent systematic review described 210 patients with a Kommerell’s diverticulum (mean age 42 years) and reported a ruptured diverticulum and aortic dissection as presentation in 4 and 11% of the patients, respectively [3]. Interestingly, in this population the vast majority (84%) of the patients underwent surgical intervention [3]. The size of the Kommerell’s diverticulum ranged from 15 to 80 mm (mean 38 ± 18 mm) in the overall population, from 20 to 60 mm in the ruptured cases and 25 to 70 mm in those with an aortic dissection [3]. Backer et al. showed that patients who undergo surgical repair because of a symptomatic Kommerell’s diverticulum (*n* = 20) had more frequently a right-sided aortic arch (*n* = 16), probably because this configuration is more likely to lead to symptomatic esophageal or tracheal enclosure [19]. Due to increased shear stress, it is hypothesized that the curvature of a right-sided aorta and a larger absolute size of the Kommerell’s diverticulum may be associated with an increased risk of rupture [20]. The potential role of primary vascular pathology or premature atherosclerosis in this population remains to be established.

To conclude, Kommerell’s diverticulum is characterized by an aneurysmatic onset (the diverticulum) of an aberrant left or right subclavian artery. The configuration of a Kommerell’s diverticulum from an aberrant left subclavian artery from a right-sided aorta is often associated with some degree of tracheal and esophageal compression, and this may result in a range of associated symptoms. Although frequently an incidental finding in asymptomatic adults, one should be aware of the intricate anatomic characteristics and potential vascular complications of such diverticulum and its presence should always warrant further investigation. Serial follow-up is warranted in cases with small dimensions and no symptoms, however, surgical intervention should be considered when patients become symptomatic, or if the diameter exceeds 30 mm in the absence of symptoms, or when there is rapid growth. Based on the current literature, defining patients at risk of acute and potentially life-threatening vascular complications remains a challenge.

## Figures and Tables

**Table 1:** An overview of the anatomical and clinical characteristics of patients 1–7.

	Age at Diagnosis (Years)	Gender	Anatomy	Compression	Dimensions of KD	Symptoms	Management
Patient 1	40	Male	Right-sided arch, aberrant LSA and KD	Mild tracheal compression	22 × 20 mm, 319 mm^2^	Incidental finding	Structural imaging follow-up
Patient 2	40	Male	Right-sided arch, aberrant LSA and KD	Partial tracheal compression	22 × 21 mm, 357 mm^2^	Incidental finding	Structural imaging follow-up
Patient 3	50	Female	Right-sided arch, aberrant LSA and KD	Symptomatic tracheal and esophageal compression	30 × 29 mm, 870 mm^2^	Dysphagia and dyspnea	Surgical resection
Patient 4	55	Male	Right-sided arch, aberrant LSA and KD	Mild tracheal compression	25 × 23 mm, 575 mm^2^	Mild dyspnea, although excellent objective exercise capacity	Structural imaging follow-up
Patient 5	63	Male	Right-sided arch, aberrant LSA and KD	Asymptomatic esophageal compression	24 × 19 mm, 343 mm^2^	Incidental finding	Structural imaging follow-up
Patient 6	23	Male	Right-sided arch, mirror imaging branching and KD	Mild tracheal and esophageal compression	27 × 21 mm, 440 mm^2^	Dyspnea, although excellent objective exercise capacity	Structural imaging follow-up
Patient 7	25	Female	Right-sided arch, aberrant LSA and KD	Mild tracheal and esophageal compression	19 × 21 mm, 399 mm^2^	Incidental finding	Structural imaging follow-up

LSA, left subclavian artery; KD, Kommerell’s diverticulum.

## Data Availability

All data relevant to the study are included in the article or uploaded as supplementary information. Additional data are available on reasonable request.
